# Activities of colistin- and minocycline-based combinations against extensive drug resistant *Acinetobacter baumannii *isolates from intensive care unit patients

**DOI:** 10.1186/1471-2334-11-109

**Published:** 2011-04-27

**Authors:** Wang Liang, Xiao-fang Liu, Jun Huang, De-mei Zhu, Jian Li, Jing Zhang

**Affiliations:** 1 Institute of Antibiotics, Huashan Hospital, Fudan University, Shanghai, China; 2 Facility for Anti-infective Drug Development and Innovation, Drug Delivery, Disposition and Dynamics, Monash Institute of Pharmaceutical Sciences, Monash University, Melbourne, Australia

## Abstract

**Background:**

Extensive drug resistance of *Acinetobacter baumannii *is a serious problem in the clinical setting. It is therefore important to find active antibiotic combinations that could be effective in the treatment of infections caused by this problematic 'superbug'. In this study, we analyzed the *in vitro *activities of three colistin-based combinations and a minocycline-based combination against clinically isolated extensive drug resistant *Acinetobacter baumannii *(XDR-AB) strains.

**Methods:**

Fourteen XDR-AB clinical isolates were collected. The clonotypes were determined by polymerase chain reaction-based fingerprinting. Susceptibility testing was carried out according to the standards of the Clinical and Laboratory Standards Institute. Activities of drug combinations were investigated against four selected strains and analyzed by mean survival time over 12 hours (MST_12 h_) in a time-kill study.

**Results:**

The time-kill studies indicated that the minimum inhibitory concentration (MIC) of colistin (0.5 or 0.25 μg/mL) completely killed all strains at 2 to 4 hours, but 0.5×MIC colistin showed no bactericidal activity. Meropenem (8 μg/mL), minocycline (1 μg/mL) or rifampicin (0.06 μg/mL) did not show bactericidal activity. However, combinations of colistin at 0.5×MIC (0.25 or 0.125 μg/mL) with each of the above were synergistic and shown bactericidal activities against all test isolates. A combination of meropenem (16 μg/mL) with minocycline (0.5×MIC, 4 or 2 μg/mL) was synergitic to all test isolates, but neither showed bactericidal activity alone. The MST_12 h _values of drug combinations (either colistin- or minocycline-based combinations) were significantly shorter than those of the single drugs (*p *< 0.01).

**Conclusions:**

This study indicates that combinations of colistin/meropenem, colistin/rifampicin, colistin/minocycline and minocycline/meropenem are synergistic in vitro against XDR-AB strains.

## Background

Nosocomial infections caused by drug-resistant *Acinetobacter baumannii *are a major global problem [1]. Recent data from the Chinese Network for Bacterial-resistance Surveillance demonstrated that the susceptibility of *A. baumannii *to carbapenems has decreased to below 50%, and almost 17% of these isolates are resistant to all antimicrobial agents that are routinely used in clinical practice [2]. Unfortunately, no new antibiotics will be available for extensive drug resistant (XDR) gram-negative pathogens including *A. baumannii *for at least a decade [3]. These data highlight the importance and urgency of finding antibiotic combinations that are effective in the treatment of infections caused by this problematic "superbug".

"Old" antibiotics, such as colistin [4] and minocycline [5], are generally active against *A. baumannii *strains. Colistin was withdrawn from general clinical use in the late 1970s because of potential nephrotoxicity and neurotoxicity, but now it is increasingly used worldwide as the last-line therapy against *A. baumannii *[6-8]. Colistin activity can be enhanced when combined with some other antibiotics with different modes of action such as carbapenems, rifampicin and ceftazidime [9-13]. Minocycline is a second-line antibiotic for a number of common bacterial infections in the clinical setting. Recent studies have indicated that minocycline is a promising drug for the treatment of *A. baumannii *infections [5,14]. Furthermore, it can enhance the activity of colistin against multidrug-resistant *A. baumannii *isolates [15].

In order to find the combinations that would achieve complete killing or are bactericidal against XDR-AB isolates, we examined the activities of colistin- and minocycline-based combinations, including colistin/meropenem, colistin/rifampicin, colistin/minocycline and minocycline/meropenem, against XDR-AB isolates from patients in the intensive care unit (ICU) of Huashan Hospital, a 1,400-bed hospital in eastern China.

## Methods

### Strains and antibiotics

Fourteen XDR-AB strains (resistant to all antimicrobial agents but one or two) [16] were isolated from patients admitted to the ICU of Huashan Hospital between January 2009 and March 2010. All these strains were identified with the API 20NE system (BioMérieux, Marcy l'Etoile, France). Clonotypes of the strains were determined by polymerase chain reaction (PCR)-based fingerprinting with primers of ERIC-1 and 2 [17]; the PCR conditions were as follows: 94°C for 2 min, followed by 40 cycles of 94°C for 1 min, 26°C for 1 min, and 72°C for 2 min, with a final extension at 72°C for 5 min.

Colistin sulfate (Lot: 30327-200305; 22106 units/mg), meropenem (Lot: 130506-200702; purity: 87%) and rifampicin (Lot: 130496-200702; purity: 98%) were obtained from the National Institute for the Control of Pharmaceutical and Biological Products of China (Shanghai, China). Minocycline hydrochloride (Lot: 2000131970/C; purity: 84%) was purchased from Wyeth Inc. (Madison, NJ, USA). Stock solutions of colistin, meropenem, rifampicin and minocycline were prepared with sterile distilled water and kept at -80°C for a maximum of 2 months before use.

### Susceptibility testing

Minimum inhibitory concentrations (MICs) of colistin, minocycline, rifampicin and meropenem against the clinical isolates were tested using the broth microdilution method according to the Clinical and Laboratory Standards Institute) protocol [18]. Briefly, serial 2-fold dilutions of an antibacterial agent in cation-adjusted Mueller-Hinton broth (CaMHB) were prepared in a 96-well microplate with an inoculum of approximately 5×10^5 ^cfu/mL. After 24-hour incubation at 35°C in ambient air, the lowest concentration with no visible bacterial growth was defined as the MIC. *Escherichia coli *ATCC25922 was used as a quality control strain. The experiments were repeated on 2 different days.

### Static time-kill studies

Four strains from different colonotypes (*A. baumannii *09-95, 09-1769, 09-2092 and 10-548) were selected for the time-kill study. Two sets of time-kill studies were conducted for each strain. In the first set, the colistin concentration was fixed at 0.5×MIC (0.25 or 0.125 μg/mL) and each of the other agents was combined with colistin from high to low concentrations as follows: meropenem, 16, 8 and 4 μg/mL; minocycline, 2, 1 and 0.5 μg/mL; and rifampicin, 0.25, 0.125 and 0.06 μg/mL. In the second set, minocycline concentrations were fixed at 0.5×MIC (4 or 2 μg/mL), and combined with meropenem at concentrations of 16 and 8 μg/mL. A growth control was included in both sets.

An overnight culture was diluted with prewarmed CaMHB to about 1×10^5 ^cfu/mL and further incubated at 35°C for 2 hours. Then the medium was divided into a series of tubes containing different concentrations of drugs. A growth control was included. All tubes were incubated in a shaking incubator (150 rpm) at 35°C. Samples were taken at 0, 2, 4, 6, 8, 10, 12 and 24 hours and viable bacteria were counted on Mueller-Hinton agar (MHA) plates. The plates were incubated for 18 hours at 35°C, and then the number of colonies formed was counted. The theoretical lower limit of quantification was 10 cfu/mL. Bactericidal activity and synergy were defined as ≥ 3 log_10 _decrease or ≥ 2 log_10 _decrease, respectively, in cfu/mL between the combination and its most active antibiotic alone [19].

### Statistical and mathematical analysis

The bactericidal effects of each drug or drug combination were quantified by calculation of the mean survival time over 12 h (MST_12 h_) as previously described [20]:

where AUC_0-12 h _was the area under the time-kill curve over 0-12 hours, AUMC_0-12 h _was the area under the time-kill curve multiplied by the time of sampling in hours from 0 to 12. Both AUMC_0-12 h _and AUC_0-12 h _were calculated using the linear trapezoidal method.

The analysis of variance (ANOVA) and *post hoc *analysis were performed to compare the differences between drug treatments and strains on MST_12 h _using SPSS statistical software (SPSS Inc., USA).

## Results

The PCR fingerprints demonstrated that the strains belonged to four clonotypes termed clonotype I, II, III and IV (Table 1). The MICs of colistin, minocycline, meropenem and rifampicin are summarized in Table 1. All isolates were resistant to meropenem but susceptible to colistin and minocycline. The MICs of rifampicin ranged from 4 to 16 μg/mL.

**Table 1:** *Acinetobacter baumannii *isolates in this study and their MICs

Isolates	Infection	Sample	Clonotype	COL (μg/mL)	MEM (μg/mL)	MINO (μg/mL)	RFP (μg/mL)
09-95	VAP	Sputum	I	0.25	32	4	4
09-154	VAP	Sputum	I	0.25	16	4	8
09-1769	VAP	Sputum	II	0.5	64	8	4
09-1782	VAP	Sputum	I	0.5	16	4	8
09-2092	VAP	Sputum	I	0.25	128	8	4
09-2221	VAP	Sputum	I	0.5	64	4	4
09-2902	CB	Sputum	III	0.5	16	4	4
10-39	CB	Sputum	I	0.5	64	2	8
10-161	CB	Sputum	I	0.25	64	2	4
10-415	II	CSF	I	0.5	64	2	4
10-533	VAP	Sputum	I	0.25	16	4	8
10-548	HAP	Sputum	IV	0.5	64	4	16
10-584	VAP	Sputum	IV	0.5	64	4	8
10-617	CB	Sputum	IV	0.5	32	2	8

MIC = minimum inhibitory concentration, VAP = ventilator-associated pneumonia, HAP = hospital-acquired pneumonia, CB = colonized bacteria, II = intracranial infection, CSF = cerebrospinal fluid, COL = colistin (breakpoint = 2 μg/mL), MEM = meropenem (breakpoint = 16 μg/mL), MINO = minocycline (breakpoint = 16 μg/mL), RFP = rifampicin (no breakpoint is available).

**Figure 1:**
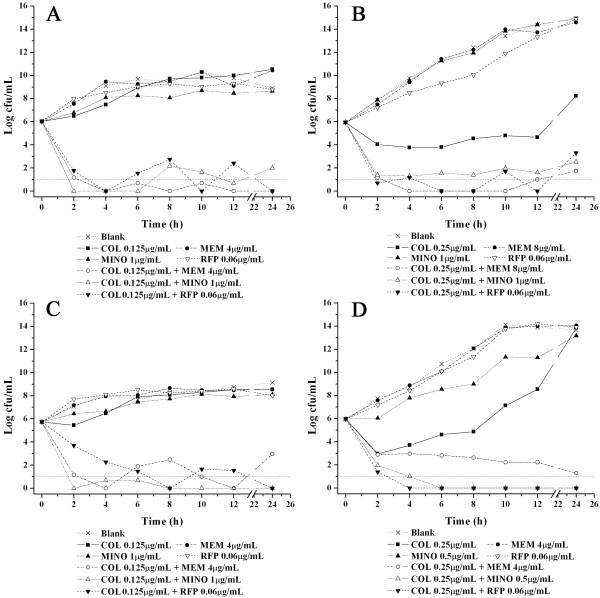
**Time-kill curves of each antibiotic alone and in combination with colistin**. (A) Isolate 09-95; (B) isolate 09-1769; (C) isolate 09-2092; (D) isolate 10-548. Dashed line (Log cfu/mL = 1) was the lower limit reference line.

In the time-kill study, no viable cells were detected at 2 to 4 hours in the presence of 1×MIC colistin, and regrowth was not observed at 24 hours (data not shown). When the concentration of colistin was 0.5×MIC, the growth of isolates 09-1769 and 10-548 was markedly reduced (Figure 1B and 1D), but the growth of isolates 09-95 and 09-2092 was only slightly inhibited compared with the controls (Figure 1A and 1C). An important phenomenon was observed in that complete killing of all strains was achieved within 2-6 hours when high concentrations of meropenem (16 μg/mL), minocycline (2 μg/mL) or rifampicin (0.25 μg/mL) were applied in combination with 0.5×MIC colistin. When combined with colistin, the lowest concentrations of minocycline, meropenem and rifampicin presenting a synergistic effect were identified for different strains (Figure 1A, B, C and 1D). For meropenem, 4 μg/mL (i.e., 1/8-1/32 MIC) was sufficient to present a synergistic effect with colistin against three strains (not isolate 09-1769) which represent 92% of the total strains. Similarly, a minocycline concentration as low as 1 μg/mL (i.e., 1/4-1/8 MIC) also showed synergy with colistin against all four isolates. The lowest concentration of rifampicin of 0.06 μg/mL also exhibited a synergistic effect with colistin (no combination of rifampicin at a concentration of less than 0.06 μg/mL with colistin was included in present work).

**Figure 2:**
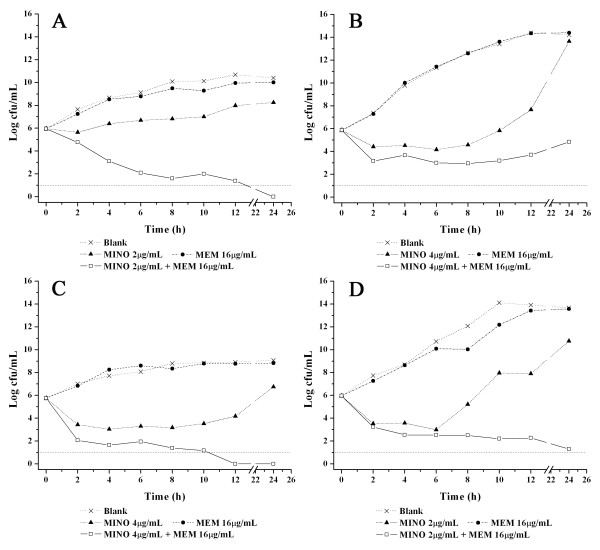
**Time-kill curves of the combinations of minocycline and meropenem**. (A) Isolate 09-95; (B) isolate 09-1769; (C) isolate 09-2092; (D) isolate 10-548. Dashed line (Log cfu/mL = 1) was the lower limit reference line.

In the time-kill study with minocycline and meropenem alone, the growth of all isolates was inhibited by 0.5×MIC minocycline, but not affected by 16 μg/mL meropenem alone (Figure 2). Interestingly, synergistic activity was observed when meropenem was combined with minocycline. A combination of 16 μg/mL meropenem with 0.5×MIC minocycline exhibited bactericidal activities against all strains at 12 h (Figure 2). Isolate 09-95 showed susceptibility to this combination with 8 μg/mL meropenem (data not shown).

**Figure 3:**
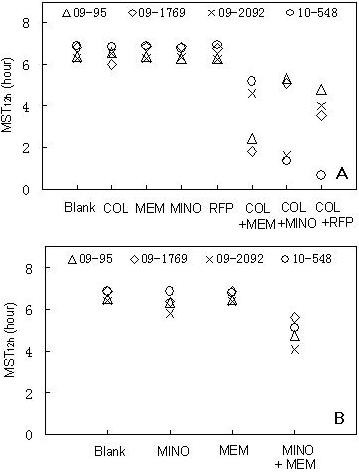
**MST_12 h _analysis**. (A) MST_12 h _of colistin-based drug combinations and each single drug; (B) MST_12 h _of the minocycline/meropenem combination and each drug alone. Concentrations of antibiotics are shown in Figure 1 and 2. MST_12 h _= mean survival time over 12 hours.

The mean survival times of each XDR-AB strain under different drug treatments are shown in Figure 3. The data indicated that MST_12 h _values of colistin- or minocycline-based combinations were significantly shorter than that of each drug alone. On average, the MST_12 h _values of colistin-based drug combinations were 3.1 hours shorter than any constituent drug alone (Figure 3A) (*p *< 0.01). The MST_12 h _value of the minocycline/meropenem combination was 1.6 hours shorter than that of minocycline or meropenem alone (Figure 3B) (*p *< 0.01).

## Discussion

Colistin is unavailable in China, so data are limited here regarding colistin in the treatment of infections caused by multidrug-resistant gram-negative bacteria. The 14 XDR-AB strains used in this study were isolated from patients in the ICU in Huashan Hospital, where there is a high incidence of severe infections and a major presence of XDR-AB. We demonstrated that colistin was active against all the strains at a lower MIC (MIC_90 _= 0.5 μg/mL) than in other reports [21]. This may arise from the fact that colistin has not yet been approved for clinical use in China. The results of the study were in accordance with previous reports that colistin/meropenem [9], colistin/rifampicin [10] and colistin/minocycline [15] combinations were synergistic against these strains.

It was considered that colistin should be combined with other drugs for lower dose-related toxicity, and recent research has suggested that it is necessary to combine colistin with another antibiotic to obtain a pharmacological effect. The most frequently used form of colistin is colistin methanesulfonate (CMS), which is a prodrug of colistin without activity [22]. *In vivo*, CMS is hydrolyzed to produce the active colistin. Pharmacokinetic studies have indicated that the recommended intravenous dose of CMS will present a stable level of colistin in plasma of 1-4 μg/mL [23], which should have activity against most *A. baumannii *strains (the MIC is 0.5-1 μg/mL). However, if protein-binding is considered [24], the concentration of free drug is likely to be reduced to 0.3-1.2 μg/mL, i.e., the concentration of colistin is under the MIC in many cases, which may result in treatment failure and increase the risk of drug resistance. To, combine it with meropenem (≥ 8 μg/mL), minocycline (≥ 1 μg/mL) or rifampicin (≥ 0.06 μg/mL) may achieve bactericidal effects according to our results. Further observations are warranted in light of these data.

As in some recent reports [5,14,15], our study also demonstrated that minocycline is generally active against *A. baumannii*. Here, the MIC of minocycline for the 14 strains was 4 μg/mL. Because of dose-dependent gastrointestinal and vestibular function disorders [25] and the risk of drug resistance, minocycline alone is generally considered unfavorable for the treatment of XDR-AB, but it may have promising effects in combination with other antibiotics. In addition to the colistin/minocycline combination, our results also showed that minocycline combined with meropenem was effective against XDR-AB isolates. Our findings point to a possible option for treatment of XDR-AB infections when colistin or CMS is unavailable.

## Conclusion

Our research indicates that combinations such as colistin/meropenem, colistin/rifampicin and colistin/minocycline are synergistic in vitro against XDR-AB strains. The minocycline/meropenem combination may be an alternative option for treatment of infections caused by XDR *A. baumannii *when colistin is unavailable.

## Competing interests

The authors declare that they have no competing interests.

## Authors' contributions

All authors read and approved the final manuscript. WL supervised the study, performed the susceptibility testing and static time-kill studies, and wrote the manuscript. XFL and JH contributed to the susceptibility testing and static time-kill studies. DMZ provided advice on the technology. JL discussed the data and helped in polishing the manuscript. JZ planned and supervised the experiments.

## Pre-publication history

The pre-publication history for this paper can be accessed here:

http://www.biomedcentral.com/1471-2334/11/109/prepub
